# Mindfulness meditation use in Britain during the COVID-19 pandemic

**DOI:** 10.1371/journal.pone.0303349

**Published:** 2024-05-13

**Authors:** Otto Simonsson, Stephen D. Fisher

**Affiliations:** 1 Department of Neurobiology, Care Sciences and Society, Karolinska Institute, Stockholm, Sweden; 2 Department of Sociology, University of Oxford, Oxford, United Kingdom; St John’s University, UNITED STATES

## Abstract

**Objectives:**

The objectives of this study were to examine the prevalence and associations of mindfulness meditation use and also its perceived mental health effects during the COVID-19 pandemic.

**Methods:**

Using repeated cross-sectional data from broad online samples weighted to be representative of the adult population in Britain, we estimated the prevalence of mindfulness meditation use and employed logistic regression models to investigate sociodemographic and political associations of mindfulness meditation use and also its perceived mental health effects during the COVID-19 pandemic.

**Results:**

The findings suggest that 16 percent of adults in Britain had learnt to practice mindfulness in 2021. In covariate-adjusted regression models, having learnt to practice mindfulness was more common among young and middle-aged adults, residents in London, and respondents who voted for the Liberal Democrats. Among mindfulness meditation users who reported having practiced mindfulness during the COVID-19 pandemic, 60 percent reported that it positively affected their mental health and 24 percent reported that it negatively affected their mental health. Notably, 41 percent of respondents with children under 18 (versus 13 percent of those without minors) reported negative mental health effects. In covariate-adjusted regression models, negative mental health effects from mindfulness practice during the COVID-19 pandemic were not concentrated in any particular groups, except for respondents with children under 18.

**Conclusions:**

Mindfulness meditation has become widespread in Britain, but the results in this study suggest that mindfulness meditation use may be concentrated in certain sociodemographic and political groups. The results also suggest that practicing mindfulness during the COVID-19 pandemic had positive mental health effects for a majority of users, but approximately one-quarter of users reported negative mental health effects. It is therefore important for future research to continue monitoring the prevalence of mindfulness meditation use in society and to investigate under what circumstances, for whom, and in what ways mindfulness-based practices may have negative effects on mental health.

## Introduction

During the COVID-19 pandemic, the British government instituted three nationwide lockdowns, which had noticeable effects on psychological distress [1, 2]. For example, in one national, longitudinal cohort study, levels of psychological distress in Britain increased following the first nationwide lockdown, with some of the greatest increases among younger adults and people living with young children [3]. Such findings highlight the importance of investigating accessible and cost-effective interventions that may counter negative mental health effects in the event of future pandemics.

The evidence to date suggests that interventions designed to cultivate mindfulness (i.e., present-moment awareness with an attitude of curiosity, openness, and acceptance) can promote mental health in normal circumstances [4], but recent studies suggest that such interventions may also have had certain mental health benefits during the quarantines and lockdowns of the COVID-19 pandemic [5, 6]. For instance, a randomized controlled trial found that an eight-week mindfulness course, compared with a waitlist control condition, reduced anxiety among university students in Britain during the third nationwide lockdown [7]. These findings suggest that mindfulness-based interventions may be effective in countering possible increases in psychological distress following pandemic-related quarantines or lockdowns. However, relatively little remains known about whom mindfulness-based interventions benefits most and why, especially during national emergencies such as the COVID-19 pandemic. It is therefore important to conduct population-based studies on the associations of mindfulness meditation use and its perceived mental health effects, which can inform future hypotheses that can be tested in randomized controlled trials.

While several population-based studies have investigated mindfulness meditation use in the United States [8, 9], only one such study has assessed mindfulness meditation use in Britain [10]. The results showed that an estimated 15 percent of adults had learnt to practice mindfulness in 2018 and that using an app was the most common way of learning it. In covariate-adjusted regression models, higher levels of engagement with mindfulness meditation was more likely among young and middle-aged adults while awareness of mindfulness meditation was more likely among female adults, unmarried adults, adults from middle and high-income households, and Remain voters in the 2016 Brexit Referendum [10]. The study was conducted before the COVID-19 pandemic, however, and also did not investigate associations of mindfulness meditation use or its perceived mental health effects, which could have been useful to better understand the potential accessibility and impact of mindfulness meditation use across sociodemographic and political groups.

Here, using repeated cross-sectional data from broad online samples weighted to be representative of the national population in Britain, we investigated the prevalence and associations of mindfulness meditation use and also its perceived mental health effects during the COVID-19 pandemic.

## Materials and methods

From 2^nd^ to 6^th^ December, 2021, an online cross-sectional survey was carried out by Deltapoll (https://deltapoll.co.uk/), a British polling company, of a sample of adults in Britain aged 18 and above. The data was weighted to be representative of the adult population of Britain. The data and syntax can be accessed at https://doi.org/10.6084/m9.figshare.25709889.v1. This was a secondary analysis of already collected data that cannot be traced back to identifiable individuals and therefore did not require ethics approval. The collection and analysis methods complied with the terms and conditions of the data source.

### Measures

#### Mindfulness-related variables

All participants were asked about their experience of mindfulness meditation and were presented with the following response options: 1) I have learnt how to practise mindfulness from a course, book, app, or other source; (2) I have heard of mindfulness meditation, have not practised it, but I am interested in it; (3) I have heard of mindfulness meditation, have not practised it, and I am not interested in it; (4) I have never heard of mindfulness meditation; (5) Don’t know. The same question and response options were used in the most recent study on mindfulness meditation use in Britain [10].

If respondents reported that they had learnt how to practice mindfulness, they were asked how they had learnt to practice mindfulness, with the following response options: 1) Attending a course; 2) Reading a book; 3) Watching a video or DVD; 4) Visiting a website; 5) Using an app; 6) Some other way; 7) Don’t know. The order of the options was randomized, except for option 6 and 7 which were fixed. The same question and response options were used in the most recent study on mindfulness meditation use in Britain [10].

Respondents who reported that they had learnt how to practice mindfulness were also asked about their experience of mindfulness meditation during the COVID-19 pandemic. The following response options were presented: 1) I practised mindfulness, and it positively affected my mental health; 2) I practised mindfulness, and it negatively affected my mental health; 3) I practised mindfulness, and it had no noticeable effect on my mental health; 4) I did not practise mindfulness during the COVID-19 pandemic; 5) Don’t know.

#### Sociodemographic and political variables

The dataset contained the same sociodemographic and political variables that were used in the most recent study on mindfulness meditation use in Britain [10]: age, gender, region of residence, education, marital status, family composition, employment status, household income, as well as voting behaviour in the Brexit Referendum on the 23^rd^ June 2016 and the General Election on the 12^th^ December 2019. However, it is important to note that the most recent study on mindfulness meditation use in Britain–carried out from 26^th^ to 27^th^ November 2018 –asked respondents about voting behavior in the General Election on the 8^th^ June 2017 [10].

### Statistical analyses

We used bivariate and multiple logistic regression models to evaluate associations between mindfulness-related variables and sociodemographic and political variables. The data from the previous study on mindfulness meditation in Britain [10] was used for comparisons between 2018 and 2021, but it was also used to increase statistical power in regression models, if the dependent variable was present in both survey years. The variables were exactly the same as those used in the previous study on mindfulness meditation in Britain [10], except for the general election variable, the variable on perceived mental health effects of mindfulness meditation use during the COVID-19 pandemic, and the survey year variable.

We used two multiple logistic regression models to evaluate associations between having learnt to practice mindfulness and sociodemographic and political variables. Model 1 only included sociodemographic variables: gender, age, region, education, marital status, family composition, employment status, household income, and survey year. Model 2 included sociodemographic and political variables: gender, age, region, education, marital status, family composition, employment status, household income, survey year, general election, and Brexit referendum. Equivalent models were run to evaluate associations between negative mental health effects from mindfulness practice and sociodemographic and political variables, though without the survey year variable.

## Results

### Frequency distributions

Table 1 presents the descriptive statistics on awareness and experience of mindfulness meditation in Britain, 2018 and 2021. As seen in the table, the percentage of respondents in 2021 who reported having learnt to practice mindfulness (16 percent) was not significantly different from the 15 percent recorded in 2018.

**Table 1:** Awareness and experience of mindfulness in Britain, 2018 and 2021.

	2018	2021
	% [95% CI]	% [95% CI]
*I have learnt how to practise mindfulness from a course*, *book*, *app*, *or other source*	15 [12, 18]	16 [13,19]
*I have heard of mindfulness meditation*, *have not practised it*, *but I am interested in it*	31 [28, 35]	27 [23, 30]
*I have heard of mindfulness meditation*, *have not practised it*, *and I am not interested in it*	25 [21, 29]	29 [25, 32]
*I have never heard of mindfulness meditation*	23 [19, 26]	19 [16, 22]
*Don’t know*	6 [5, 8]	10 [7, 12]

Note: The number of observations was 1,013 for 2018 and 1,859 for 2021. The percentages were weighted to reflect the sociodemographic profile of the adult population of Britain and were rounded to the closest integer.

Table 2 presents the descriptive statistics on pathways to learning to practise mindfulness in Britain, 2018 and 2021. As shown in the table, the most common way of learning to practise mindfulness was the same across both years: using an app.

**Table 2:** Pathways to learning to practice mindfulness in Britain, 2018 and 2021.

	2018	2021
	% [95% CI]	% [95% CI]
*Attending a course*	24 [16, 34]	17 [12, 23]
*Reading a book*	34 [24, 45]	30 [22, 38]
*Watching a video or DVD*	17 [10, 27]	21 [15, 29]
*Visiting a website*	15 [10, 22]	33 [25, 42]
*Using an app*	35 [25, 47]	38 [29, 48]
*Some other way*	13 [7, 22]	15 [9, 24]
*Don’t know*	0 [0, 0]	0 [0, 2]

Note: The number of observations for each item (i.e., the number of respondents who had learnt to practice mindfulness) was 153 in 2018 and 402 in 2021. The percentages were weighted to reflect the sociodemographic profile of the adult population of Britain and were rounded to the closest integer. The total amounts to more than 100 percent, as the respondents could tick more than one option.

Table 3 presents the response distribution of people’s experience of mindfulness meditation in Britain during the COVID-19 pandemic, a question that was asked only of those who had learnt to practise mindfulness. As demonstrated in the table, the majority of mindfulness meditation users (90 percent) reported having practiced mindfulness during the pandemic. Of those, 60 percent reported that it positively affected their mental health and 24 percent reported that it negatively affected their mental health. The difference in perceived mental health effects was noticeable, for example, among those who had children under 18 and those who did not (48 vs 69 percent positive, 41 vs 13 percent negative; see S1 Table).

**Table 3:** Mindfulness practice in Britain during the COVID-19 pandemic.

	2021
	% [95% CI]
*I practised mindfulness*, *and it positively affected my mental health*	54 [44, 63]
*I practised mindfulness*, *and it negatively affected my mental health*	22 [16, 30]
*I practised mindfulness*, *and it had no noticeable effect on my mental health*	14 [9, 20]
*I did not practise mindfulness during the COVID-19 pandemic*	8 [3, 17]
*Don’t know*	2 [1, 10]

Note: The number of observations was 402. The percentages were weighted to reflect the sociodemographic profile of the adult population of Britain and were rounded to the closest integer.

### Logistic regressions

#### Having learnt to practice mindfulness

Table 4 presents the results from the regressions of having learnt to practice mindfulness, with the data from 2018 and 2021 combined into a single dataset. As seen in the table, in all models, having learnt to practice mindfulness was more common among young and middle-aged adults, residents in London, and respondents who voted for the Liberal Democrats.

**Table 4:** Logistic regression models–Having learnt to practice mindfulness (2018 and 2021).

	Bivariate		Model 1		Model 2	

VARIABLES	Coef	Se	Coef	Se	Coef	Se
Gender (Male)						
Female	-0.08	(0.16)	0.04	(0.17)	0.04	(0.17)
Age (55 or more)						
18–34	1.31***	(0.25)	0.98**	(0.32)	0.91**	(0.32)
35–55	0.96***	(0.25)	0.66*	(0.31)	0.61*	(0.31)
Region (Midlands)						
Scotland	0.18	(0.29)	0.36	(0.30)	0.34	(0.33)
Wales	-0.63	(0.35)	-0.58	(0.37)	-0.58	(0.39)
London	0.67**	(0.25)	0.60*	(0.26)	0.61*	(0.26)
North	-0.35	(0.25)	-0.24	(0.25)	-0.22	(0.25)
South	0.14	(0.25)	0.28	(0.26)	0.31	(0.26)
Education (Degree)						
No Degree	-0.27	(0.15)	-0.06	(0.17)	-0.05	(0.18)
Marital Status (Married)						
Not Married	0.16	(0.17)	-0.16	(0.20)	-0.21	(0.19)
Family Composition (No Children under 18)						
Children under 18	0.57***	(0.17)	0.29	(0.18)	0.25	(0.18)
Employment Status (Working)						
Unemployed	-0.03	(0.26)	-0.05	(0.27)	-0.12	(0.28)
Retired	-1.24***	(0.28)	-0.52	(0.40)	-0.56	(0.39)
Student	0.63	(0.38)	0.42	(0.38)	0.39	(0.39)
Stay-at-home parent/housekeeper	-0.71	(0.40)	-0.72	(0.40)	-0.74*	(0.37)
Household Income (£28,000 or less)						
£28,001-£55,000	-0.03	(0.18)	-0.17	(0.19)	-0.15	(0.19)
£55,001 or more	0.16	(0.23)	-0.15	(0.25)	-0.12	(0.25)
Survey year (2018)						
2021	0.08	(0.16)	0.08	(0.16)	0.09	(0.16)
2017 and 2019 General Elections (Conservative)						
Labour	0.57**	(0.20)	…..	…..	0.41	(0.22)
Liberal Democrats	0.80*	(0.38)	…..	…..	0.87*	(0.44)
Other	0.82**	(0.26)	…..	…..	0.58	(0.30)
Did not vote	0.95***	(0.21)	…..	…..	0.55	(0.29)
2016 Brexit Referendum (Leave)						
Remain	0.10	(0.18)	…..	…..	-0.33	(0.19)
Did not vote	0.42	(0.23)	…..	…..	-0.18	(0.29)
Observations	2,872		2,872		2,872	
Pseudo R2	…..		0.0642		0.0737	
Intercept	…..		-1.97		-2.15	

Robust standard errors in parentheses

*** p≤0.001

** p≤0.01

* p≤0.05

Note: The bivariate analyses regress the dependent variable on a single predictor (including categorical predictor variables). Although the coefficients from these models are shown in one column, they come from separate regressions. The 2021 dataset was combined with the 2018 dataset and survey year was controlled for in the multiple logistic regressions. See S1 File for additional analyses.

#### Mindfulness practice and negative effects on mental health

Table 5 presents the results from the regressions of negative effects on mental health from mindfulness practice, which only included respondents who reported having practiced mindfulness during the COVID-19 pandemic. As shown in the table, in all models, negative mental health effects from mindfulness practice was more common among respondents with children under 18.

**Table 5:** Logistic regression models–Mindfulness practice negatively affected mental health (2021).

	Bivariate		Model 1		Model 2	

VARIABLES	Coef	Se	Coef	Se	Coef	Se
Gender (Male)						
Female	0.09	(0.43)	-0.16	(0.40)	-0.20	(0.39)
Age (55 or more)						
18–34	1.58*	(0.63)	1.14	(0.86)	1.17	(0.90)
35–55	0.89	(0.65)	0.23	(0.88)	0.28	(0.92)
Region (Midlands)						
Scotland	0.59	(0.76)	0.75	(0.89)	1.15	(0.89)
Wales	1.32	(0.79)	2.03*	(0.93)	1.96	(1.06)
London	0.27	(0.57)	0.34	(0.71)	0.01	(0.75)
North	-0.20	(0.62)	0.10	(0.82)	-0.10	(0.87)
South	0.02	(0.71)	0.40	(0.81)	0.10	(0.84)
Education (Degree)						
No Degree	-0.27	(0.43)	0.08	(0.41)	0.12	(0.42)
Marital Status (Married)						
Not Married	-0.28	(0.44)	-0.26	(0.53)	-0.22	(0.56)
Family Composition (No Children under 18)						
Children under 18	1.53***	(0.40)	1.43**	(0.48)	1.48**	(0.47)
Employment Status (Working)						
Unemployed	-1.20	(0.72)	-0.94	(0.80)	-1.34	(0.95)
Retired	-1.11	(0.85)	0.41	(1.17)	0.37	(1.15)
Student	1.32	(0.84)	1.30	(0.86)	1.30	(0.84)
Stay-at-home parent/housekeeper	-0.74	(0.93)	-1.40	(0.87)	-1.76	(1.01)
Household Income (£28,000 or less)						
£28,001-£55,000	-0.08	(0.42)	-0.26	(0.48)	-0.19	(0.45)
£55,001 or more	0.65	(0.64)	0.03	(0.72)	0.18	(0.69)
2019 General Election (Conservative)						
Labour	0.23	(0.57)	…..	…..	-0.24	(0.51)
Liberal Democrats	-0.73	(0.89)	…..	…..	-0.22	(0.93)
Other	-0.29	(0.69)	…..	…..	-1.31	(0.71)
Did not vote	0.07	(0.53)	…..	…..	-0.83	(0.74)
2016 Brexit Referendum (Leave)						
Remain	0.18	(0.49)	…..	…..	0.09	(0.42)
Did not vote	0.36	(0.51)	…..	…..	0.82	(0.76)
Observations	376		376		376	
Pseudo R2	…..		0.1591		0.1761	
Intercept	…..		-1.18		-0.83	

Robust standard errors in parentheses

*** p≤0.001

** p≤0.01

* p≤a0.05

Note: The bivariate analyses regress the dependent variable on a single predictor (including categorical predictor variables). Although the coefficients from these models are shown in one column, they come from separate regressions. Regressions only include respondents who reported having practiced mindfulness during the COVID-19 pandemic.

## Discussion

The present study used data from broad online samples weighted to be representative of the adult population of Britain to investigate the prevalence and associations of mindfulness meditation use and also its perceived mental health effects during the COVID-19 pandemic. The results suggest that 16 percent of adults in Britain had learnt to practice mindfulness in 2021, compared with 15 percent in 2018 [10]. In covariate-adjusted regression models, having learnt to practice mindfulness was more common among residents in London and respondents who voted for the Liberal Democrats. It was also more common among young and middle-aged adults, which corresponds with results from a population-based study on mindfulness meditation use in the United States [8]. Such findings suggest that older adults may have higher barriers (e.g., psychological, technological, cultural dimensions) to mindfulness meditation than younger age groups. Future research should investigate this possibility further.

Among those who reported mindfulness meditation use during the COVID-19 pandemic, 60 percent reported that it had positive mental health effects, but 24 percent reported negative mental health effects, which broadly corresponds with findings from other survey studies [11]. For example, in two survey studies with samples of meditators who had at least two months of meditation experience, approximately one-quarter of the respondents reported previous unwanted or unpleasant experiences related to meditation practice [12, 13]. Another study, with a sample representative of the US adult population with regards to age, sex and ethnicity, found that approximately 32 percent of respondents with any meditation experience reported having had challenging, difficult, or distressing experiences as a result of their meditation practice [9]. Although the sample populations and the items presented to respondents (e.g., adverse effects, unwanted effects) differed across studies, the results in this study contribute to a growing body of evidence suggesting that meditation may not exclusively be associated with mental health benefits. Future research on potential risks associated with meditation practice should utilize standardized measures to make it easier to compare results across studies.

Notably, in covariate-adjusted analyses, respondents with children under 18 were more likely to report negative mental health effects during the COVID-19 pandemic. Previous studies suggest that parental psychological distress was high during the pandemic and the resulting school closures [14], especially among those with young children [3]. Although research suggests that mindfulness training may reduce parenting stress [15], it is possible that mindfulness meditation use during the pandemic was counterproductive for some parents. If specific groups experience negative effects of a certain intervention, it is important to investigate why this might be the case. Future research should use qualitative research methods to better understand the experience of mindfulness meditation during the COVID-19 pandemic among parents with underaged children.

The study design had several strengths and limitations which should be considered while interpreting the results. First, given that the samples were weighted to be representative of the adult population of Britain, reliable population estimates could be produced in the analyses. The samples may have been too small, however, to detect significant differences on several variables in the regression models, especially in the subsample of respondents who reported mindfulness meditation use during the COVID-19 pandemic (n = 376). To maintain statistical power and the representativeness of the samples, unclear and uncommon responses (e.g., don’t know, prefer not to say) were not coded as missing values (see S1 File), but sensitivity analyses showed no major difference to the main results with other coding. Second, the use of sociodemographic and political variables allowed for estimates on how mindfulness meditation use and its perceived mental health effects were distributed across groups in society. There were no psychological variables included, though, which could have been used to better understand potential mechanisms underlying mindfulness meditation use and its perceived mental health effects. Third, the item on perceived mental health effects of mindfulness meditation use during the COVID-19 pandemic did not capture the degree to which or the way in which effects were positive or negative. The respondents were also not presented with a specific definition of mindfulness practice, which may have caused various interpretations among respondents of what mindfulness practice means. Future research should continue monitoring the prevalence of mindfulness meditation use in society and use other research designs (e.g., qualitative, longitudinal) and statistical methods (e.g., machine learning) to examine under what circumstances, for whom, and in what ways mindfulness-based practices may have negative effects on specific aspects of mental health (e.g., anxiety, depression, stress).

## Supporting information

S1 Table Children versus no children. (DOCX)

S1 File (DOCX)
