# Supplementary material for: Mindfulness meditation use in Britain during the COVID-19 pandemic
Source: PLoS One. 2024 May 13;19(5):e0303349. doi: 10.1371/journal.pone.0303349 (PMC11090315; doi:10.1371/journal.pone.0303349)
Supplement: S1 Table — (DOCX) [file pone.0303349.s001.docx]

**S1 Table**

| S1 Table. Children versus No Children | | |
| --- | --- | --- |
|  | Children | No Children |
|  | % [95% CI] | % [95% CI] |
| *I practised mindfulness, and it positively affected my mental health* | 48 [34, 61] | 69 [57, 79] |
| *I practised mindfulness, and it negatively affected my mental health* | 41 [28, 55] | 13 [8, 21] |
| *I practised mindfulness, and it had no noticeable effect on my mental health* | 12 [6, 20] | 18 [10, 29] |
| Note: The number of observations for those who had practiced mindfulness during the COVID-19 pandemic was 376. The percentages were weighted to reflect the sociodemographic profile of the adult population of Britain and were rounded to the closest integer. | | |
