# Supplementary material for: Mindfulness meditation use in Britain during the COVID-19 pandemic
Source: PLoS One. 2024 May 13;19(5):e0303349. doi: 10.1371/journal.pone.0303349 (PMC11090315; doi:10.1371/journal.pone.0303349)
Supplement: S1 File — (DOCX) [file pone.0303349.s002.docx]

**S1 File**

***Exploratory analyses***

In further analysis we tested for all possible interaction effects between time and the different explanatory variables in our model to check whether there were substantial changes in the patterns of reporting having learnt to practice mindfulness. All but two of the 24 interaction terms were statistically insignificant. The two that were significant do suggest that reporting having learnt to practice mindfulness has become relatively more prevalent among Remain voters (p = .024) and among those who live in the South of England (p = .013). However, we should not declare them as findings and they should not be over-interpreted, as they might be accidental given the total number of tests conducted and given that the overall level of mindfulness use has not changed.

***Coding of variables***

**Gender**

*Which of the following best describes how you think of yourself?*

The respondents had the following options: (1) ‘Male’; (2) ‘Female’; (3) ‘In another way’; (4) ‘Prefer not to say’. The answers were coded into two subcategories: (1) ‘Male’ and (2, 3, 4) ‘Female’.

**Age**

*And how old are you?*

The respondents gave a numerical response. The answers were coded into three subcategories: ‘Young Adults’ (18-34 years), ‘Middle-Aged Adults’ (35-54 years), and ‘Older Adults’ (55+ years).

**Region of Residence**

*Where do you live?*

The respondents had the following options: (1) Scotland; (2) North-West; (3) North-East; (4) Yorkshire & Humberside; (5) Wales; (6) West Midlands; (7) East Midlands; (8) South-West; (9) South-East; (10) Eastern; (11) London. The answers were coded into six subcategories: (1) ‘Scotland’, (5) ‘Wales’, (11) ‘London’, (2, 3, and 4) ‘North’, (6 and 7) ‘Midlands’, and (8, 9, and 10) ‘South’.

**Education**

*What is the highest educational level that you have achieved?*

The respondents had the following options: (1) Secondary school; (2) University degree or equivalent professional qualification, NVQ level 4; (3) Higher university degree, doctorate, MBA, NVQ level 5 etc; (4) Still in full time education; (5) No formal education; (6) Don't know/prefer not to answer. The answers were coded into two subcategories: (1, 5, and 6) ‘No Degree’ and (2, 3, and 4) ‘Degree’.

**Household Income**

*What is the combined annual income of your household, prior to tax being deducted?*

The respondents had the following options: (1) £7,000 or less; (2) £7,001-£14,000; (3) £14,001-£21,000; (4) £21,001-£28,000; (5) £28,001-£34,000; (6) £34,001-£41,000; (7) £41,001-£48,000; (8) £48,001-£55,000; (9) £55,001-£62,000; (10) £62,001-£69,000; (11) £69,001-£76,000; (12) £76,001-£83,000; (13) £83,001 or more; (14) Prefer not to say. The answers were coded into three subcategories: 1-4 and 14) ‘Less than £28,000’, (5-8) ‘£28,001-£55,000’, and (9-13) ‘More than £55,001’.

**Employment Status**

*Which of the following best describes your working status?*

The respondents had the following options: (1) Working full time – working 30+ hours per week school; (2) Working part time – working between 8-29 hours per week; (3) Not working but seeking work or sick; (4) Not working and not seeking work; (5) Retired on a state pension only; (6) Retired with a private pension; (7) Student; (8) Stay at home parent or housekeeper; (9) Prefer not to answer. The answers were coded into five subcategories: (1, 2, and 9) ‘Working’, (3 and 4) ‘Not Working’, (5 and 6) ‘Retired’, (7) ‘Student’, and (8) ‘Stay at home parent or housekeeper’.

**Marital Status**

*Which of the following best describes your marital status?*

The respondents had the following options: (1) Prefer not to answer; (2) Single; (3) Married; (4) Civil partnership; (5) Co-habiting; (6) Widowed; (7) Separated; (8) Divorced. The answers were coded into two subcategories: (1-2 and 4-8) ‘Not Married’ and (3) ‘Married’.

**Family Composition**

*Do you have any children aged 18 or under?*

The respondents had the following options: (1) No children aged 18 or under; (2) Yes – children aged under 5 years old; (3) Yes – children aged 5-10 years old; (4) Yes – children aged 11-15 years old; (6) Yes – children aged 16-18 years old; (7) Prefer not to answer. The answers were coded into two subcategories: (1 and 7) ‘No children aged 18 or under’, (2-6) ‘Children aged 18 or under’.

**2016 Brexit Referendum Voting**

*In the Referendum on the 23rd of June 2016 on whether the UK should remain in or leave the European Union which way did you vote? - or did you not vote? Please select only one answer.*

The respondents had the following options: (1) Voted to Leave the EU; (2) Voted to Remain in the EU; (3) Did not vote; (4) Don’t remember; (5) Prefer not to say. The answers were coded into three subcategories: (1) ‘Leave’, (2) ‘Remain’, and (3, 4, 5) ‘Did not vote’.

**2017 General Election Voting**

*Which party did you vote for in the General Election on June 8^th^ 2017?*

The respondents had the following options: (1) Conservative; (2) Labour; (3) Liberal Democrat; (4) Scottish National Party (SNP); (5) Plaid Cymru (PC); (6) UK Independence Party (UKIP); (7) Green; (8) Some other party; (9) Don’t remember; (10) Prefer not to say; (11) Did not vote. The answers were coded into five subcategories: (1) ‘Conservatives’, (2) ‘Labour’, (3) ‘Lib Dems’, (4, 5, 6, 7, 8, 9, and 10) ‘Other’, and (.) ‘Did not vote’.

**2019 General Election Voting**

*Which party did you vote for in the General Election on December 12^th^ 2019?*

The respondents had the following options: (1) Conservative; (2) Labour; (3) Liberal Democrat; (4) Scottish National Party (SNP); (5) Plaid Cymru (PC); (6) Brexit Party; (7) UK Independence Party (UKIP); (8) Green; (9) Some other party; (10) Don’t remember; (11) Did not vote; (12) Refuse. The answers were coded into five subcategories: (1) ‘Conservatives’, (2) ‘Labour’, (3) ‘Lib Dems’, (4, 5, 6, 7, 8, 9, 10, 12) ‘Other’, and (11 and .) ‘Did not vote’.

**2017 and 2019 General Election Voting**

For the regressions of having learnt to practice mindfulness, the 2017 and 2019 General Election Voting variables were combined with the following coding: ‘Conservatives’, ‘Labour’, ‘Lib Dems’, ‘Other’, and ‘Did not vote’.
